# IL-21 Promotes Late Activator APC-Mediated T Follicular Helper Cell Differentiation in Experimental Pulmonary Virus Infection

**DOI:** 10.1371/journal.pone.0105872

**Published:** 2014-09-24

**Authors:** Jae-Kwang Yoo, Thomas J. Braciale

**Affiliations:** 1 Inflammation Research, Amgen Inc., Seattle, Washington, United States of America; 2 Beirne B. Carter Center for Immunology Research, University of Virginia, Charlottesville, Virginia, United States of America; 3 Department of Microbiology, University of Virginia, Charlottesville, Virginia, United States of America; 4 Department of Pathology, University of Virginia, Charlottesville, Virginia, United States of America; McGill University, Canada

## Abstract

IL-21 is a type-I cytokine that has pleiotropic immuno-modulatory effects. Primarily produced by activated T cells including NKT and T_FH_ cells, IL-21 plays a pivotal role in promoting T_FH_ differentiation through poorly understood cellular and molecular mechanisms. Here, employing a mouse model of influenza A virus (IAV) infection, we demonstrate that IL-21, initially produced by NKT cells, promotes T_FH_ differentiation by promoting the migration of late activator antigen presenting cell (LAPC), a recently identified T_FH_ inducer, from the infected lungs into the draining lymph nodes (dLN). LAPC migration from IAV-infected lung into the dLN is CXCR3-CXCL9 dependent. IL-21-induced TNF-α production by conventional T cells is critical to stimulate CXCL9 expression by DCs in the dLN, which supports LAPC migration into the dLN and ultimately facilitates T_FH_ differentiation. Our results reveal a previously unappreciated mechanism for IL-21 modulation of T_FH_ responses during respiratory virus infection.

## Introduction

Following infection with pathogenic microorganisms, the encounter of B cells with their cognate specific Ag in secondary lymphoid organs triggers B cell activation, proliferation and differentiation ultimately resulting in germinal center (GC) formation within B cell follicles. The GC response is particularly pronounced due to the inflammatory stimulus produced by the invading microorganisms. GC B cell responses and GC formation is largely T cell dependent. Hallmarks of the GC response include BcR affinity maturation, plasma cell differentiation and the generation of memory B cells. Hence, the GC response not only contributes to pathogen clearance but also plays a pivotal role in preventing subsequent infections with the infecting microorganism [1]–[5]. T_FH_ T cells are recently recognized as a distinct CD4^+^ T cell subset defined as PD1^+^CXCR5^+^Bcl-6^+^. This T-cell subset has been implicated as a key regulator of the GC B cell response through the delivery of multiple soluble and cell-associated signals to GC B cells including the production of soluble factors (IL-4 and IL-21) and the display of co-stimulatory ligands and receptors (ICOS, CD28, CD40L and CD84) [4], [6]–[10].

The factors controlling T_FH_ differentiation are not as yet fully understood, and multiple cell types and molecules have been implicated in this process [4], [6]. IL-21 was initially proposed as a key soluble factor driving the differentiation of Ag-primed CD4^+^ T cells along the T_FH_ lineage pathway [8], [11], and is now recognized as promoting an optimal T_FH_ response [12], [13]. However, the mechanism(s) by which IL-21 optimizes the T_FH_ response has not as yet been clearly defined.

Recently, we have identified a novel immune cell population in virus infected murine lungs with migratory properties and antigen presenting capacity, the late activator antigen presenting cell (LAPC) [14]. The mPDCA1^+^CD11c^−^B220^−^TcRβ^−^ LAPCs initiate their migration out of the IAV-infected lungs into the draining lymph nodes relatively late in the course of infection (i.e., between 6–12 days post-infection (d.p.i.)) *via* CXCR3-CXCL9 dependent chemotactic pathway. In the dLN, LAPCs promote T_FH_ differentiation of Ag-activated CD4^+^ T cells by display of ICOSL and engagement of ICOS receptor on the activated CD4^+^ T cells [14]–[16]. In this report we demonstrate that IL-21, initially produced by NKT cells, promotes optimal T_FH_ differentiation by augmenting CXCR3-CXCL9 dependent LAPC migration into the dLN during influenza A virus (IAV) infection. IL-21-induced TNF-α production by conventional T cells is critical to stimulate CXCL9 expression by DCs in the dLN, which supports LAPC migration into the dLN and ultimately facilitates T_FH_ differentiation.

## Materials and Methods

### Mice, virus and infections

CD45.1^+^ or CD45.2^+^ C57BL/6 mice were purchased from National Cancer Institute (NCI). *Tnf-α* ^−/−^ mice were generated in the Ludwig Institute for Cancer Research, purchased from Taconic farms and bred in house. *Il-21rα* ^−/−^, *il-21* ^−/−^, and OT-II mice were bred in house. *Cd-1d* ^−/−^ mice were provided by M.D. Okusa (University of Virginia, Charlottesville, VA). All mice were housed in a specific pathogen–free environment and all mouse experiments were performed in accordance with protocols approved by the University of Virginia Animal Care and Use Committee. A/WSN/OVA-II virus was generously provided by Dr. David Topham (University of Rochester, Rochester, USA) [18]. For virus infection, mice were infected intranasally (i.n.) with a sub-lethal dose (0.05 LD_50_) of influenza strain A/PR/8/34 (H1N1), A/WSN/33 (H1N1) or A/WSN/OVA-II in serum-free Iscove’s medium, after anesthesia with ketamine and xylazine.

### Quantitative RT-PCR

dLN cell suspensions were prepared as described [14], [15]. DCs were isolated by FACS (Reflection HAPS 2) to examine *cxcl-9* expression. mRNA isolation, reverse transcription and real-time PCR were performed as previously described [19]. Data were generated with the comparative threshold cycle method, by normalizing to hypoxanthine phosphoribosyltransferase (*hprt*). The sequences of primers used in the studies are available on request.

### Bone marrow chimeras

To generate mixed bone marrow (BM) chimeras containing wild type (CD45.1^+^) and *il21-rα ^−/−^* (CD45.2^+^) BM in a 1∶1 ratio, we lethally irradiated (1,100 rads) CD45.1^+^ wild type B6 mice and reconstituted the irradiated mice with CD45.1^+^ wild type BM (2×10^6^ cells) mixed with CD45.2^+^ *il-21rα ^−/−^* BM (2×10^6^ cells). After 8 weeks, using PBMC the reconstitution efficiency was determined by FACS-analysis and the successfully reconstituted mice were then infected with A/PR/8/34 IAV.

### OT-II T cell transfer, infection and *ex vivo* co-culture with LAPCs

For OT-II T cell transfer into CD45.1^+^ wild type B6 mice, cells were isolated from CD45.2^+^ OT-II lymph nodes (LNs). A total of 5×10^6^ LN cells were then transferred into CD45.1^+^ mice by *i.v.* injection. The recipient mice were infected with A/WSN/OVA-II virus 24 hrs later. At 5 d.p.i., *in vivo* virus activated OT-II cells were isolated from the dLN by FACS. LAPCs were sorted separately at 8 d.p.i. from the dLNs of A/WSN/OVAII infected either wt or *il-21rα* ^−/−^ mice. Isolated day 5 *in vivo* virus activated OT-II cells were *ex vivo* co-cultured with day 8 LAPC for additional 24 hrs to assess T_FH_ differentiation by FACS-analysis.

### Cell sorting

For *ex vivo* co-culture experiments, recipients of transferred OT-II T-cells or wild type mice were infected with A/WSN/OVA-II influenza. Different cell populations from the dLN were sorted by FACS (Reflection HAPS 2) based on the following markers at either 5 or 8 d.p.i.: OT-II cells, CD45.2^+^Thy1.2^+^CD4^+^; LAPCs, mPDCA1^+^CD11c^−^B220^−^TcRβ^−^. For *cxcl-9* qPCR, DCs (CD11c^+^TcRβ^−^) were sorted from the dLN of A/PR/8/34 IAV infected wild type mice at 6 d.p.i. For *tnf-α* qPCR, both wild type (CD45.1^+^) and *il-21rα* ^−/−^ (CD45.2^+^) T cells (CD4 and CD8 T) were sorted by FACS from the dLN of A/PR/8/34 IAV-infected mixed BM chimera mice at 6 d.p.i. (CD45.1^+^Thy1.2^+^CD4^+^, CD45.2^+^Thy1.2^+^CD4^+^, CD45.1^+^Thy1.2^+^CD8^+^, CD45.2^+^Thy1.2^+^CD8^+^). For *in vivo* adoptive transfer experiments, non-T_FH_ total T cells (Thy1.2^+^CXCR5^−^) were isolated by FACS from the dLN of A/PR/8/34 IAV-infected wild type or *tnf-α^−/−^* mice at 6 d.p.i. and adoptively transferred by the *i.v.* route (2×10^6^cells/mouse) into 6 d.p.i. A/PR/8/34 IAV-infected recipient *tnf-α^−/−^* mice.

### Antibodies and FACS-analysis

All antibodies were purchased from BD Biosciences or eBioscience (unless otherwise stated): CD4 (L3T4), CD8α (53-6.7), CD11c (HL3), CD45.1 (A20), CD45.2 (104), CD90.2 (30-H12), B220/CD45R (RA3-6B2), NK1.1 (PK136), TCR-β (H57-597), IL-21 (FFA21), CXCR5 (2G8), PD-1 (RMP1-30), ICOS-L (HK5.3), TNF-α (MP6-XT22) and CXCL9 (MIG-2F5.5). αmPDCA-1 mAb (JF05-1C2.4.1) was purchased from Miltenyi Biotec. A CXCR3 specific mAb was obtained from both R&D Systems (220803) and Biolegend (CXCR3-173). αmTNF-α mAb (XT3.11) were purchased from BioXcell for *in vivo* mTNF-α blocking experiments. Flow cytometry was performed on FACS-Canto with optimal compensation set for six-color staining. The data were analyzed using FlowJo software (Tree Star, Inc.). All cytokine (IL-21 and TNF-α) and chemokine (CXCL9) expressions by dLN-derived cells were measured directly *ex vivo* without further *in vitro* re-stimulation.

### *In vivo* migration assay

Both B6 and *il-21rα* ^−/−^ mice were anesthetized as described above and infected by i.n. instillation with 50 µl PBS containing 0.05 LD_50_ A/PR/8/34 virus. On day 5 p.i., mice received 50 µl of either PBS (negative control) or FITC-Dextran (40 kDa, 1 mg/ml) by i.n. instillation. At 24 hrs post-treatment, mice were sacrificed and cells were collected from the dLN. FACS-analysis was performed to examine the percent population of FITC^+^ cells, gating on LAPCs (mPDCA-1^+^CD11c^−^B220^−^TCRβ^−^).

### *In vivo* TNF-α blocking experiments

To examine the role of TNF-α in LAPC-mediated T_FH_ differentiation, *in vivo* TNF-α blocking experiments were performed. Briefly, B6 mice infected with 0.05LD_50_ A/PR/8/34 virus were treated (i.p.) daily with either isotype control Abs (Rat IgG) or mTNF-α blocking mAb (200 µg/day/mouse) from 4 d.p.i. till 7 d.p.i. At 8 d.p.i, the levels of CXCL9 expression in DCs, LAPC accumulation and T_FH_ differentiation in the dLN were monitored and compared between isotype Ab treated and mTNF-α blocking mAb treated mice by FACS-analysis.

### IAV–specific antibody ELISA

BAL fluid was collected from IAV–infected mice on 8 d.p.i. by intra-tracheal instillation of 500 µl of sterile PBS, and anti-influenza antibody responses in the BAL fluid were measured by ELISA. Briefly, wells of 96-well plates were coated overnight at room temperature with 50 µl of either A/PR/8 or B/Lee influenza virus. The plates were washed twice with PBS supplemented with 0.05% Tween-20 (PBST) and incubated with 50 µl of 2% BSA in PBST for 1 hr at room temperature. After washing the plates with PBST, 50 µl of diluted BAL fluid was added to each well and incubated for 2 hrs at room temperature. Bound antibodies were detected by the incubation of horseradish peroxidase (HRP)–conjugated anti–mouse IgM (1∶10,000; SouthernBiotech) or total IgG (1∶10,000; SouthernBiotech) antibodies. After 1 hr, the plates were washed with PBST, and 100 µl of 3,3′,5,5′-tetramethylbenzidine (TMB) substrate solution (Sigma-Aldrich) was added into each well and incubated for a further 30 minutes. The enzyme reaction was stopped by adding 100 µl of 2N H_2_SO_4_ and O.D. values were determined at 450 nm using a plate reader (Bio-TEK).

### Statistical analysis

Unless otherwise noted, an unpaired two-tailed Student’s *t*-test was used to compare two treatment groups. Groups larger than two were analyzed with one-way ANOVA (Tukey’s post-test). These statistical analyses were performed using Prism3 software (for Macintosh; GraphPad Software, Inc.). Data are mean ± s.e.m. A p value of <0.05 was considered to be statistically significant.

## Results

### IL-21 can promote T_FH_ differentiation in CD4^+^ T cells lacking an IL-21 receptor

**Figure 1:**
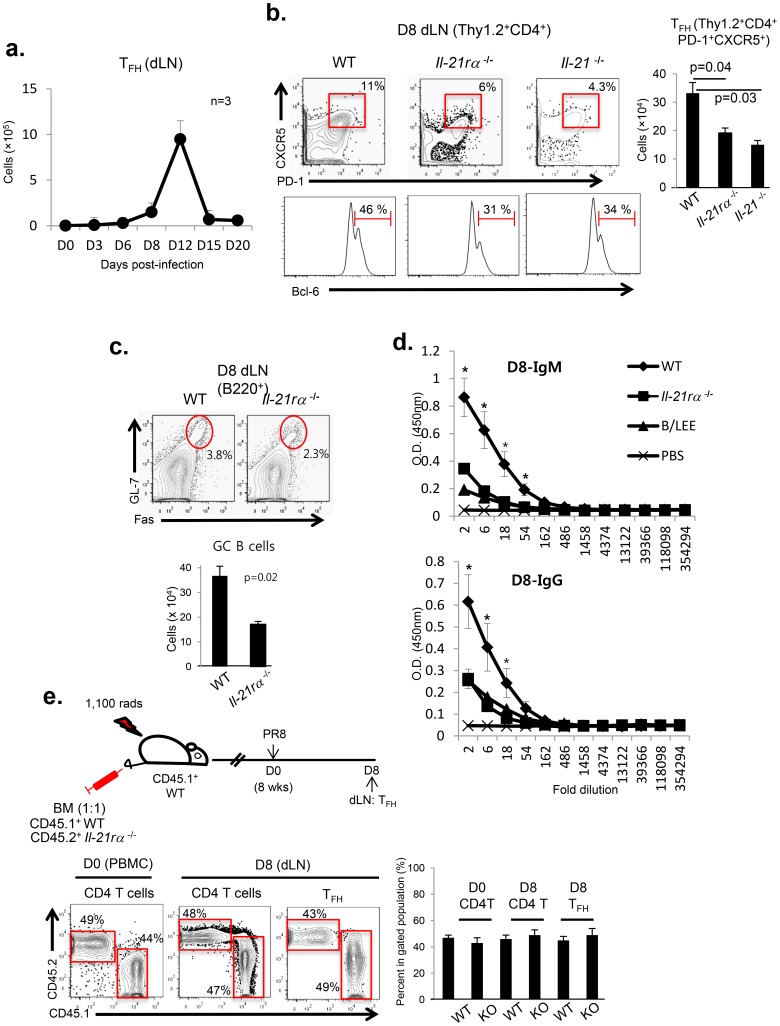
IL-21 can promote T_FH_ differentiation in CD4^+^ T cells lacking an IL-21 receptor. C57BL/6 (WT) (n = 51), *il-21rα* ^−/−^ (n = 9) and *il-21* ^−/−^ mice (n = 9) were infected intranasally (i.n.) with a sub-lethal dose (0.05 LD_50_) of A/PR/8/34 virus, as described in the Materials and Methods. (**a**) The kinetics of T_FH_ accumulation in the dLNs of B6 mice was monitored by flow cytometric (FACS) analysis. The data are presented as absolute number of T_FH_ (mean ± s.e.m.) and representative data from more than three independent experiments are shown. (**b**) The magnitude of the T_FH_ (Thy1.2^+^CD4^+^PD-1^+^CXCR5^+^ or Thy1.2^+^CD4^+^Bcl-6^+^) response in the dLNs of IAV infected B6, *il-21* ^−/−^ and *il-21rα* ^−/−^ mice was determined at 8 d.p.i. by FACS-analysis. Representative data from three independent experiments are shown. The data are presented as both percentage T_FH_ cells within the total CD4^+^ T cell population as well as absolute T_FH_ cell numbers (mean ± s.e.m.) and were analyzed by Student’s *t* test. 8 d.p.i. (**c**) GC-B cells (dLN:B220^+^Fas^+^GL7^+^, FACS) and (**d**) anti-influenza Ab responses (BALF: IgM and total IgG, Ab-ELISA) were determined. Representative data from two independent experiments are shown. Considered a significant difference at * (WT *vs.il-21rα^−/−^*, P<0.05). (**e**) Mixed BM chimera containing wild type *and il-21rα* ^−/−^ BM in a 1∶1 ratio were generated as described in the Materials and Methods. At 8 wks after reconstitution, mice (n = 7) were infected with A/PR/8/34 virus. At 8 d.p.i., the percentage of wild type (CD45.1) and *il-21rα^−/−^* (CD45.2) T-cells among total CD4^+^ T cells or T_FH_ (Thy1.2^+^CD4^+^PD1^+^CXCR5^+^) cells in the dLNs were determined by FACS-analysis. Representative images of two independent experiments are shown.

To characterize T follicular helper cell response to primary IAV infection at a mucosal tissue i.e. the respiratory tract, we examined the kinetics of generation and accumulation of T_FH_ T cells in the draining mediastinal lymph nodes (dLN) of C57BL/6 mice intra-nasally (i.n.) infected with a sub-lethal dose (0.05LD_50_) of A/PR/8/34 virus. The generation of T_FH_ T cells (i.e. CD4^+^PD1^+^CXCR5^+^Thy1.2^+^) was monitored in the dLN by FACS-analysis. As previously shown in BALB/c mice [16], in the uninfected mice the number of T_FH_ cells was negligible. T_FH_ T cells were first detected at 6 d.p.i. and showed the accumulation (absolute number) of T_FH_ cell in the dLN was maximum at 12 d.p.i. (Fig. 1a). The kinetics of T_FH_ expansion and contraction do differ modestly from that reported by Boyden et al. [20]. The most likely explanation for this difference is the virus infectious dose since Boyden et al. used 0.1 LD_50_ A/PR/8/34 virus.

To examine whether IL-21 is necessary for optimal T_FH_ differentiation in IAV infection, we evaluated the generation/accumulation of T_FH_ cells in the dLN of IAV infected *il-21rα* deficient (*il-21rα* ^−/−^), *il-21* deficient (*il-21* ^−/−^) and wild type (WT) mice. As reported previously [8], [12], [13], as early as 8 d.p.i., i.e. prior to full expansion of T_FH_ cells in the dLN, IAV-infected both *il-21rα* ^−/−^ and *il-21* ^−/−^ mice showed significantly diminished T_FH_ (PD-1^+^CXCR5^+^ or Bcl-6^+^) generation/accumulation compared to wild type mice, both in absolute T_FH_ numbers and percentage relative to other cell types (Fig. 1b). These results suggest that in IAV infection IL-21 activity may be necessary to support optimal T_FH_ differentiation. Correlated with diminished T_FH_ response, at 8 d.p.i. IAV-infected *il21rα* ^−/−^ mice exhibited significantly diminished both germinal center (GC)-B cell (Fig. 1c) and anti-IAV antibody responses (Fig. 1d) compared to wild type mice.

At present, it is uncertain whether IL-21 supports T_FH_ differentiation primarily through direct engagement of the IL-21 receptor (IL-21R) on activated proliferating CD4^+^ T cells or if other indirect mechanisms of IL-21 action also play a role [12], [21]. To address this issue during IAV infection, we constructed mixed bone marrow (BM) chimera in which lethally irradiated (1,100 rads) C57BL/6 mice (WT: CD45.1^+^) were reconstituted with a one-to-one mixture of BM from CD45.1^+^ *il-21rα* ^+/+^ and CD45.2^+^ *il21rα* ^−/−^mice. Eight weeks after the successful BM reconstitution, mice were infected with A/PR/8/34 virus and at 8 d.p.i. the abundance of wild type (CD45.1^+^) and *il-21rα* ^−/−^ (CD45.2^+^) T_FH_ in the dLN were determined (Fig. 1e). Notably, at 8 d.p.i. the ratio between wild type (CD45.1^+^) and *il-21rα* ^−/−^ (CD45.2^+^) T_FH_ was comparable to that of total CD4^+^ T cells in the dLN. Collectively, these results suggest two possibilities: 1. That during IAV infection IL-21 may support efficient T_FH_ differentiation independently of IL-21R signaling in the responding CD4^+^ T cells; 2. That the stimulus resulting from IL-21/IL21R interaction in a signaling competent cell type in the dLN e.g. T-cells, is necessary but can act in trans, which support T_FH_ differentiation of CD4^+^ T cells lacking the IL-21R receptor.

### The tempo of IL-21 production in the dLN of IAV-infected mice correlates with LAPC accumulation at this site

**Figure 2:**
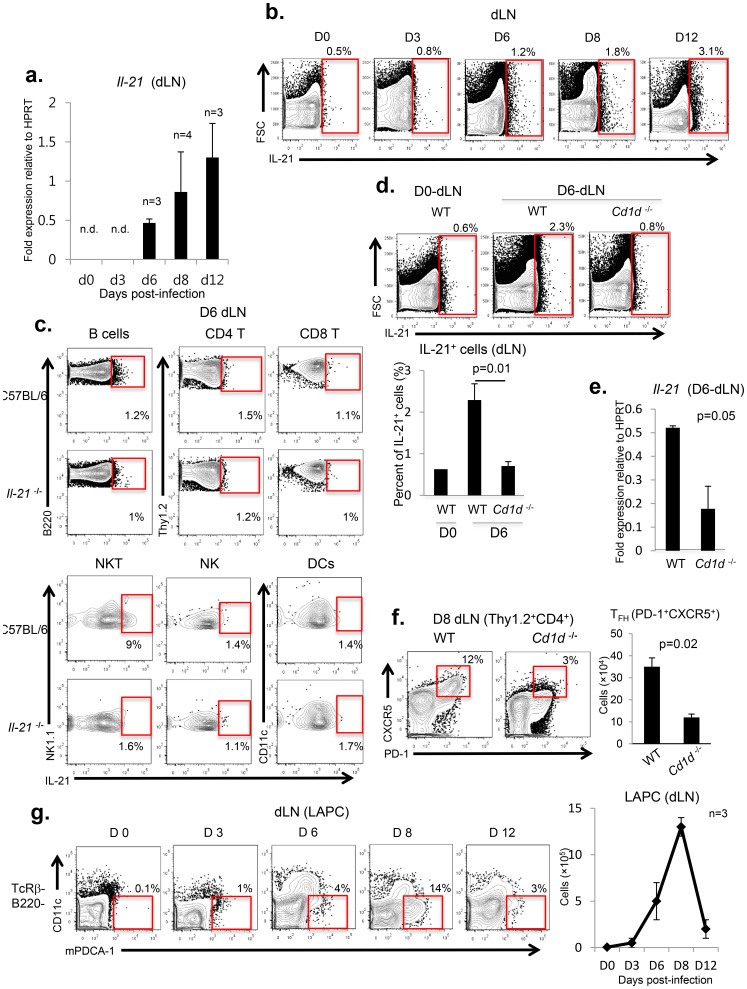
IL-21 expression and LAPC accumulation in the dLN of IAV-infected mice exhibit comparable kinetics. The tempo of IL-21 expression was examined by both (**a**) qPCR and (**b**) FACS-analysis in the dLNs of A/PR/8/34 virus infected C57BL/6 mice (n = 32). Representative data from three independent experiments are shown. (**c**) At 6 d.p.i., IL-21 expression was examined by FACS-analysis in each gated population isolated from the dLNs of C57BL/6 mice using IAV-infected *il-21* ^−/−^ mice (n = 6) as a negative control for IL-21 gating: all cells were pre-gated for live lymphocytes based on FSC/SSC profile (B cells: B220^+^ CD11c^−^Thy1.2^−^; CD4 T cells: CD4^+^Thy1.2^+^; CD8 T cells: CD8α^+^Thy1.2^+^; NKT cells: NK1.1^+^TcRβ^+^CD1d^+^PD-1^−^CXCR5^−^: NK cells: NK1.1^+^TcRβ^−^; DCs: CD11c^+^Thy1.2^−^). For CD1d-tetramer and T_FH_ marker (PD-1 and CXCR5) staining, either unloaded CD1d-tetramers, isotype control Abs (Rat IgG_2b_ :RG2b) or secondary Abs (streptavidin-APC:st-APC) has been used as negative controls for CD1d-tetramer, PD-1 or CXCR5 staining, respectively. Representative data from two independent experiments are shown. C57BL/6 (n = 6) and iNKT cell deficient *cd-1d* ^−/−^ mice (n = 6) were infected with 0.05 LD_50_ of A/PR/8/34 virus. At 6 d.p.i. the impact of NKT cell deficiency (c*d-1d* ^−/−^ mice) on IL-21 production in the dLN was examined by both (**d**) FACS-analysis and (**e**) qPCR. Representative data from two independent experiments are shown. (**f**) 8 d.p.i., T_FH_ response was determined in the dLN of both wild type and *cd-1d* ^−/−^ mice by FACS-analysis. The data are presented as both a percentage and absolute T_FH_ numbers (mean ± s.e.m.). Representative stainings from two independent experiments are shown. (**g**) C57BL/6 mice (n = 32) were infected intranasally (i.n.) with 0.05 LD_50_ of A/PR/8/34 virus, as described in the Materials and Methods. At the indicated days after infection, the extent of LAPC (mPDCA1^+^CD11c^−^B220^−^TcRβ^−^) accumulation within the dLNs was determined by FACS-analysis. The data are presented as both a percentage of lineage^−^ i.e. TcRβ^−^ B220^−^ cells and absolute LAPC numbers (mean ± s.e.m.). Representative stainings from at least three independent experiments are shown.

To further investigate the underlying mechanism accounting for the contribution of IL-21 to T_FH_ differentiation, we next examined the kinetics of IL-21 expression in the dLN of A/PR/8/34 virus infected C57BL/6 mice. Time course studies revealed that expression of IL-21 both at the gene and protein level is first detected at 6 d.p.i. and keep increasing untill 12 d.p.i., in keeping with the kinetics of T_FH_ accumulation in the dLN of IAV-infected mice (Fig. 1a, 2a and 2b).

IL-21 is primarily produced by activated T cells including NKT and T_FH_ cells [22]–[25]. We next evaluate the potential sources of IL-21 produced in the dLN of IAV-infected wt mice using IAV-infected *il-21* ^−/−^ mice as negative control for IL-21 staining. We analyzed cells for expression of IL-21 protein directly *ex vivo* from the dLN without re-stimulation *in vitro*. Interestingly, even though at 8 d.p.i. CD4^+^ T cells became major cell type expressing *il-21* gene (unpublished data), at 6 d.p.i. NKT cells (NK1.1^+^TcRβ^+^CD1d^+^PD1^−^CXCR5^−^) were most prominent cell type producing IL-21 in the dLN of IAV-infected C57BL/6 mice (Fig. 2c). This data was further confirmed using a mouse model lacking NKT cells (*cd-1d* ^−/−^ mice) showing that at 6 d.p.i. both protein and gene expressions of IL-21 in the dLNs were significantly impaired in IAV-infected *cd-1d* ^−/−^ mice compared to wild type mice (Fig. 2d and 2e). Since IL-21 promotes T_FH_ differentiation of CD4 T cells during IAV infection and NKT cells are initial primary source of IL-21 in the dLN, we determined T_FH_ response in the dLN of IAV-infected *cd1d* ^−/−^ mice and found that at 8d.p.i. IAV-infected *cd-1d* ^−/−^ mice exhibited significantly diminished T_FH_ response compared to wild type mice (Fig. 2f). Together, these data suggest that at the early phase of T_FH_ development following IAV infection NKT cells may serve as an initial major (primary) source of IL-21 in the dLN.

Recently, we have identified a novel migratory immune cell type, LAPC, in the respiratory track of IAV-infected mice [14]. LAPCs unlike conventional APCs such as respiratory dendritic cells (DCs) migrate from the infected lung tissue into the dLN late, i.e. starting at 6 d.p.i. during IAV infection and have been demonstrated to promote the differentiation of Ag-primed activated CD4^+^ T cells along the T_FH_ differentiation pathway [14]–[16]. As with IL-21 production, the kinetics of LAPC accumulation in the dLN directly parallels T_FH_ accumulation in the dLN (Fig. 1a, 2a, 2b and 2g).

### IL-21 receptor signaling modulates LAPC migration from lung tissue into the dLN of IAV-infected mice

**Figure 3:**
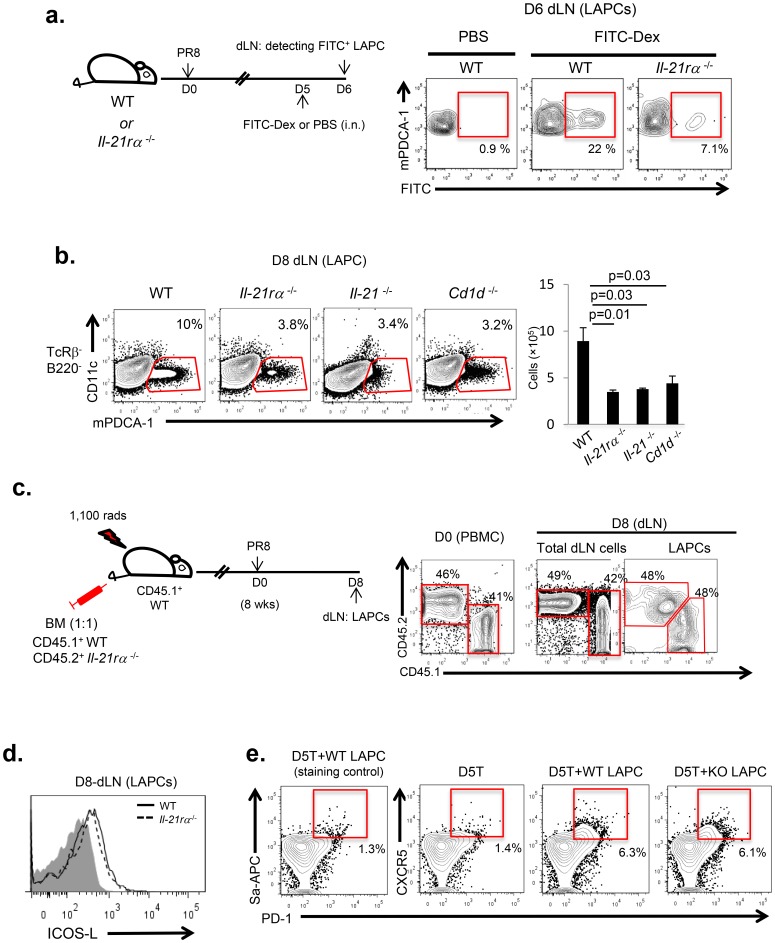
IL-21 modulates LAPC migration from lung tissue into the dLN of IAV-infected mice. (**a**) C57BL/6 (WT) (n = 12) *and il-21rα* ^−/−^ mice (n = 6) were infected with IAV, followed by FITC-Dextran administration i.n. on 5 d.p.i. 24 hrs later cells were isolated from the dLNs and the extent of LAPCs migration was determined by FACS-analysis. Numbers indicate the percentage of FITC^+^ cells within the LAPC population. Data representative of two independent experiments are shown. (**b**) At 8 d.p.i, LAPC accumulation in the dLNs was examined by FACS-analysis in C57BL/6 (WT) (n = 15), *il-21rα* ^−/−^(n = 15), *il21rα* ^−/−^ (n = 15) and *cd-1d* ^−/−^ mice (n = 6). The data are presented as both a percentage of lineage^−^ i.e. TcRβ^−^ B220^−^ cells and absolute LAPC numbers (mean ± s.e.m) and were analyzed by Student’s *t* test. Representative data of five independent experiments are shown. (**c**) The effect of IL-21R signaling on LAPC migration into the dLNs was evaluated in mixed BM chimera containing wild type (CD45.1^+^) *and il-21rα* ^−/−^ (CD45.2^+^) BM in a 1∶1 ratio as described in the Materials and Methods. At 8 wks post reconstitution, mice (n = 7) were infected with A/PR/8/34 virus. At 8 d.p.i., the percentage of wild type (CD45.1) and *il-21rα* ^−/−^ (CD45.2) LAPCs among total LAPCs (mPDCA1^+^CD11c^−^B220^−^TcRβ^−^) was determined in the dLNs by FACS-analysis. Representative images of three independent experiments are shown. (**d**) Both C57BL/6 (n = 6) *and il-21rα* ^−/−^ mice (n = 6) were infected with 0.05 LD_50_ of A/PR/8/34 virus. At 8 d.p.i., ICOSL expression on LAPCs isolated from the dLNs of C57BL/6 *and il-21rα* ^−/−^ mice was examined by FACS-analysis. The gray histogram represents isotype control Ab staining for ICOS-L. Representative data from two independent experiments are shown. (e) *In vivo* Ag primed OT-II cells (D5T) were generated as described in the Materials and Methods. FACS-sorted 5 d.p.i. OT-II cells (5×10^4^ cells/well) (D5T) were incubated with LAPCs (mPDCA1^+^CD11c^−^B220^−^TcRβ^−^) (2.5×10^4^ cells/well) isolated from the dLNs of A/WSN/OVA-II virus infected either C57BL/6 (wt) (n = 9) *and il-21rα* ^−/−^ (ko) mice (n = 18) at 8 d.p.i. 24 hrs after *ex vivo* co-culture, T_FH_ differentiation (PD-1 and CXCR5 expression) in the OT-II (CD45.2^+^Thy1.2^+^CD4^+^) T-cells was evaluated by FAC-analysis. Representative data of three independent experiments are shown.

Since in the mixed bone marrow (BM) chimera the absence of the IL-21R on the responding anti-viral CD4^+^ T cells did not diminish the generation of CD4^+^ T_FH_ T cells in the dLN but the kinetics of IL-21 expression paralleled with LAPC accumulation in the dLNs, we considered the possibility of IL- 21 expression and LAPC migration might be linked. To examine the contribution of IL-21 in the migration of LAPCs from IAV-infected lungs into the dLN, we next evaluated the migration of LAPCs following i.n. FITC-Dextran administration and uptake of this fluorescent marker by LAPCs in IAV-infected wild type and *il-21rα* ^−/−^ mice. Interestingly, *il-21rα* ^−/−^ mice showed significantly diminished FITC positive LAPC accumulation in the dLN at 6 d.p.i. compared to wild type mice (Fig. 3a). Although we cannot formally exclude the possibility that the diminished accumulation of FITC positive LAPC from *il-21rα* ^−/−^ mice in the dLN at day 5–6 p.i reflects defective uptake of FITC-dextran in the lung by LAPC deficient in IL-21R signaling, diminished LAPC accumulation in the dLN of both *il-21rα* ^−/−^ and *il-21* ^−/−^ mice in terms of absolute LAPC numbers suggests that IL-21/IL-21R signaling plays a pivotal role in the migration of LAPCs from IAV-infected lungs into the dLN (Fig. 3b). Since NKT cells are the initial primary source of IL-21 in the dLN of IAV-infected mice (Fig. 2d and 2e), the mice lacking NKT cells (*cd-1d* ^−/−^ mice) showed significantly diminished LAPC accumulation in the dLNs comparable to that of *il-21rα* ^−/−^ mice at 8 d.p.i. (Fig. 3b).

To determine if the deficit in LAPC migration in mice deficient in the IL-21 receptor was attributable to a defect in the expression of this receptor by LAPC, we constructed mixed bone marrow (BM) chimeras in which mice were reconstituted with a one-to-one mixture of BM from CD45.1^+^ *il-21rα* ^+/+^ and CD45.2^+^ *il-21rα* ^−/−^ mice. Eight weeks after BM reconstitution, mice were infected with IAV and at 8 d.p.i. the frequency of wild type (CD45.1^+^) and *il-21rα* ^−/−^ (CD45.2^+^) LAPCs in the dLN were determined (Fig. 3c). Notably, at 8 d.p.i. the ratio of wild type (CD45.1^+^) to *il-21rα* ^−/−^ (CD45.2^+^) LAPCs was comparable and equivalent to that of total dLN cells. These results suggest that IL-21 modulates LAPC migration from infected lung tissue into the dLN independently of IL-21R signaling in LAPCs.

We recently reported that ICOS-L expression by LAPC and the engagement of ICOS on CD4^+^ T cells is required for LAPC to promote T_FH_ differentiation [16]. We therefore wanted to determine whether IL-21 not only affects LAPC migration into the dLN but also directly enhances the capacity of LAPC to facilitate T_FH_ differentiation by up-regulating ICOS-L expression on LAPC. We found, however, that LAPCs isolated from the dLN of *il-21rα* ^−/−^ mice showed comparable level of ICOS-L expression to that of LAPCs from wild type mice (Fig. 3d). To further evaluate the impact of IL- 21 signaling on the ability of LAPCs to support T_FH_ differentiation, LAPC were isolated from IAV infected *il-21rα* ^−/−^ mice and co-cultured with activated CD4^+^ T cells. Briefly, OVA-specific TCR transgenic CD4^+^ OT-II T cells were isolated from naive CD45.2^+^ OT-II mice and transferred by the intra-venous (*i.v.*) route into CD45.1^+^ C57BL/6 mice. 24hrs later, mice were sub-lethally infected i.n. with the recombinant IAV A/WSN/OVA-II virus which expresses the OVA epitope recognized by OT-II cells. At 5 d.p.i., that is the time p.i. when the majority (>95%) of transferred OT-II T cells displayed an activated (CD44^hi^ or CD62L^lo^) phenotype but did not as yet express the characteristic T_FH_ phenotype (PD1^+^CXCR5^+^) [14], [16], *in vivo* activated IAV specific OT-II T cells were isolated from the dLN. These activated CD4^+^ T cells were placed in short-term (24 hrs) culture with LAPCs isolated from the dLNs of 8 d.p.i. A/WSN/OVA-II virus infected wild type or *il-21rα* ^−/−^ mice. LAPC driven T_FH_ differentiation of the OT-II T cells was monitored by flow cytometry. As shown in figure 3e, LAPCs isolated from *il-21rα* ^−/−^ mice were comparable to their wild type counterparts in promoting T_FH_ differentiation of Ag-primed CD4^+^T cells. This result further suggests that IL-21 does not modulate intrinsic capacity of LAPC to support T_FH_ differentiation.

### IL-21 enhances CXCL9 expression by DCs in the dLN of IAV-infected mice by an IL-21R independent mechanism

**Figure 4:**
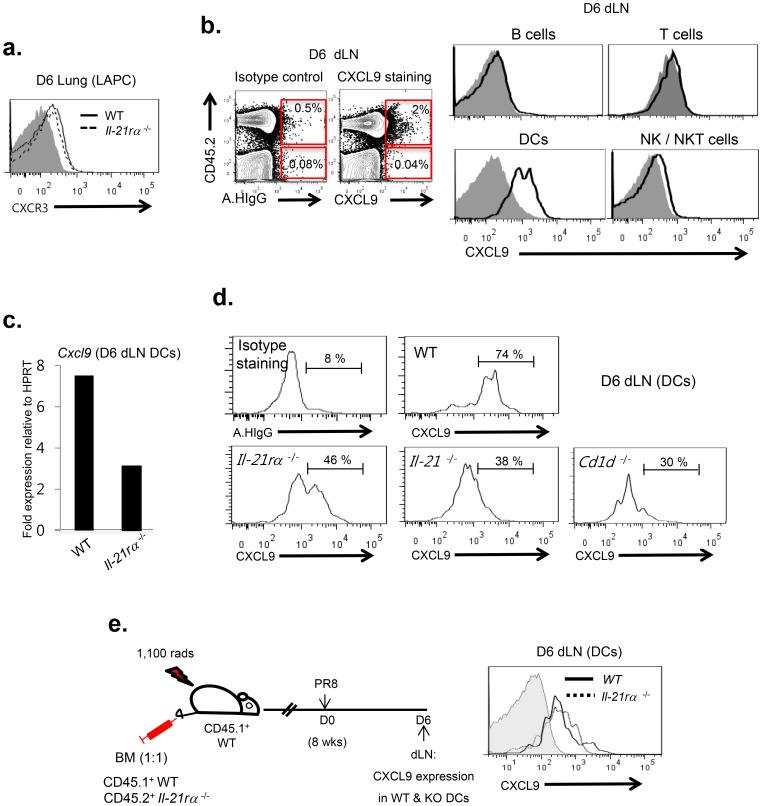
IL-21 enhances CXCL9 expression in IL-21R deficient dLN DCs following IAV infection. (**a**) C57BL/6 (WT) (n = 6) *and il-21rα* ^−/−^ mice (n = 6) were infected with 0.05 LD_50_ of A/PR/8/34 virus. At 6 d.p.i., CXCR3 expression on LAPCs isolated from the lungs of these mice was analyzed by FACS-analysis. The gray histogram represents isotype control Ab staining for CXCR3. Representative data from two independent experiments are shown. (**b**) CXCL9 expression on prominent mononuclear cell subsets was examined by FACS-analysis in each gated population (B cells: CD45.2^+^B220^+^ CD11c^−^Thy1.2^−^; T cells: CD45.2^+^Thy1.2^+^B220^−^; DCs: CD45.2^+^CD11c^+^Thy1.2^−^; NK/NKT cells: CD45.2^+^NK1.1^+^) isolated from the dLNs of 6 d.p.i. C57BL/6 mice (n = 9). The gray histogram represents isotype control Ab staining for CXCL9. Representative data from three independent experiments are shown. (**c & d**) C57BL/6 (WT) (n = 12), *il-21rα* ^−/−^ (n = 12), *il-21* ^−/−^ (n = 6) and *cd-1d* ^−/−^ mice (n = 6) were infected with A/PR/8/34 virus. (**c**) At 6 d.p.i., DCs were isolated by FACS-sorting from the dLNs of both C57BL/6 (WT) and *il-21rα* ^−/−^ mice. *Cxcl9* gene expression in these isolated DCs was evaluated by qPCR. Representative data from two independent experiments are shown. (**d**) The expression level of CXCL9 in DCs was also examined by FACS-analysis in dLN-derived DCs isolated from 6 d.p.i. C57BL/6 (WT), *il-21rα* ^−/−^, *il-21* ^−/−^ and *cd-1d ^−/−^* mice. Representative data from two independent experiments are shown. (**e**) To examine whether IL-21 modulates CXCL9 production from DCs *via* IL-21R signaling in DCs, mixed BM chimera mice established using wild type (CD45.1^+^) *and il-21rα* ^−/−^ (CD45.2^+^) BM in a 1∶1 ratio were generated as described in the Materials and Methods. At 8 wks after reconstitution, mice (n = 7) were infected with A/PR/8/34 virus. At 6 d.p.i., the expression level of CXCL9 in wild type and *il-21rα* ^−/−^ DCs (CD11c^+^Thy1.2^−^) was compared in the dLNs by FACS-analysis. Representative images of two independent experiments are shown.

LAPC in the IAV-infected lungs express CXCR3 and the migration of the cells from the lungs into the dLN is CXCL9 dependent [16]. Since IL-21 promotes LAPCs migration into the dLN, we questioned whether CXCR3 and/or CXCL9 expression was regulated by IL-21 receptor signaling during IAV infection. We observed that at the peak of LAPC accumulation in the infected lungs i.e. 6 d.p.i. when the LAPC initiate migration into the dLN [14], LAPC isolated from the lungs of infected wild type and *il21rα* ^−/−^ mice expressed CXCR3 at comparable levels (Fig. 4a). By contrast, the expression of CXCL9 in the 6 d.p.i. dLN, which is largely restricted to CD45^+^ cells primarily DC (Fig. 4b and [16]), is substantially diminished in DCs from the dLN of infected *il-21rα* ^−/−^ mice (Fig. 4c and 4d). Importantly, compared to wild type DC, CXCL9 expression was likewise decreased in DC isolated from 6 d.p.i. dLN of infected *il-21* ^−/−^ mice (Fig. 4d). IAV-infected *cd-1d^−/−^* mice, deficient for the initial primary source of IL-21, NKT cell, also showed significantly diminished expression of CXCL9 in DCs comparable to that of *il-21* ^−/−^ mice (Fig. 4d). Of note, the gene encoding c*xcl-10,* another CXCR3 ligand, was not expressed in the dLN of wild type mice during the course of IAV infection (unpublished data).

To directly address the impact of IL-21R signaling in dLN DCs on CXCL9 expression by these cells, we employed the CD45.1^+^ *il-21rα* ^+/+^ and CD45.2^+^ *il-21rα* ^−/−^ mixed bone marrow (BM) chimera strategy described above (Fig. 3). After BM reconstitution, mice were infected with IAV and at 6 d.p.i. the CXCL9 expression levels by wild type (CD45.1^+^) and *il-21rα* ^−/−^ (CD45.2^+^) DCs in the dLN were determined by FACS-analysis (Fig. 4e). We found that at 6 d.p.i. the CXCL9 expression by wild type (CD45.1^+^) and *il-21rα* ^−/−^ (CD45.2^+^) DCs were comparable. This finding suggests that IL21 regulates CXCL9 production by DCs through a mechanism independent of IL-21R signaling in the chemokine producing dLN DCs.

### IL-21 enhances TNF-α production by T cells in the dLN of IAV-infected mice

**Figure 5:**
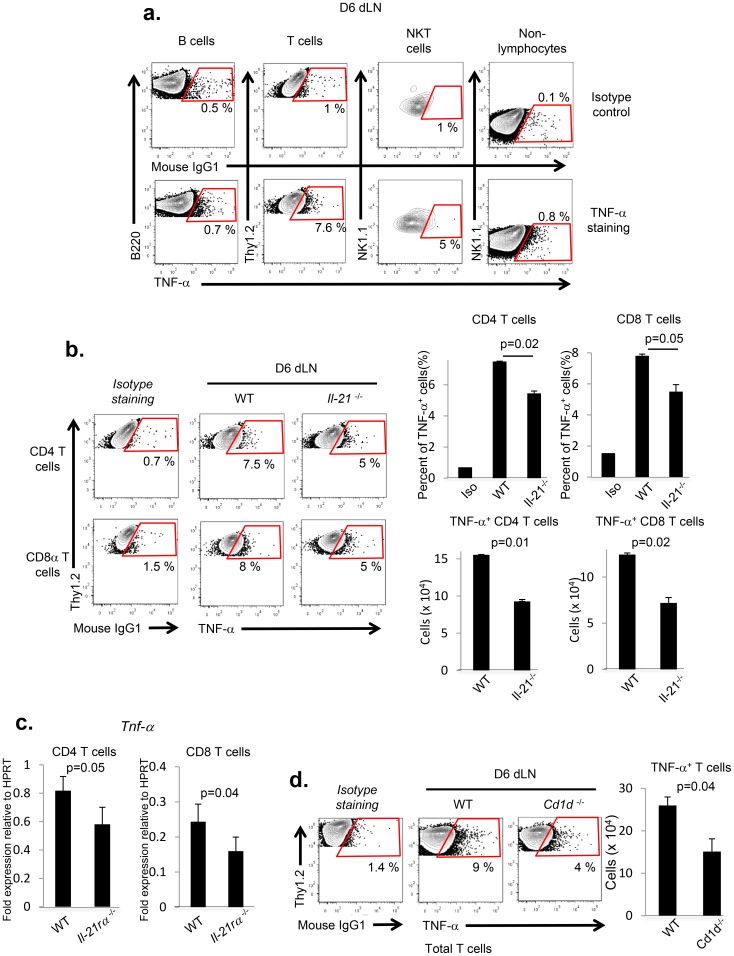
IL-21 stimulates TNF-α production by T cells in the dLN of IAV-infected mice. (**a**) C57BL/6 (n = 6) mice were infected with 0.05 LD_50_ of A/PR/8/34 virus. At 6 d.p.i., mTNF-α expression in gated lymphoid (B (B220^+^Thy1.2^−^), T (B220^−^Thy1.2^+^), NKT (NK1.1^+^TcRβ^+^CD1d^+^) and non-lymphocytes (B220^−^Thy1.2^−^) cell types isolated from the dLNs was examined by FACS-analysis. The data are representative from two independent experiments and presented as percent mTNF-α^+^ cells in the particular cell type. (**b & d**) C57BL/6 (WT) (n = 9), *il-21* ^−/−^(n = 9) and *cd-1d* ^−/−^ mice (n = 6) were infected with 0.05 LD_50_ of A/PR/8/34 virus. At 6 d.p.i., mTNF-α expression in CD4^+^ T (CD4^+^Thy1.2^+^), CD8^+^ T (CD8α^+^Thy1.2^+^) and total T cells (Thy1.2^+^) isolated from the dLNs of infected mice was examined by FACS-analysis. The data are presented as both percent of mTNF-α^+^ cells within the T-cells subset and absolute number of mTNF-α^+^ cells (mean ± s.e.m.). The data were analyzed using Student’s *t* test. Representative data from three independent experiments are shown. (**c**) Mixed BM chimeras containing wild type (CD45.1^+^) and *il-21rα ^−/−^* (CD45.2^+^) BM in a 1∶1 ratio were generated. After 8 weeks, the successfully reconstituted mice were infected with A/PR/8/34 IAV. On 6 d.p.i. both wild type (CD45.1^+^) and *il-21rα^−/−^* (CD45.2^+^) T cells (both CD4 and CD8 T cells) were isolated from the dLN by FACS (CD45.1^+^Thy1.2^+^CD4^+^, CD45.2^+^Thy1.2^+^CD4^+^, CD45.1^+^Thy1.2^+^CD8^+^, CD45.2^+^Thy1.2^+^CD8^+^) and *tnf-α* gene expression in sorted T cells was determined by qPCR. The data were analyzed using Student’s *t* test. Representative data from two independent experiments are shown.

IL-21 can modulate a variety of the immuno-regulatory functions including IL21R signaling dependent up-regulation of TNF-α production by immune cells notably activated T cells [26]–[28]. TNF-α is well recognized as a critical regulator for immune and inflammatory cell migration into tissues through its capacity to enhance the expression of a variety of chemokines including CXCL9 [29]–[31]. It was therefore of interest to determine whether TNF-α was expressed in the dLN of IAV-infected mice at 6 d.p.i. and also to identify the cell type(s) producing TNF-α. This analysis revealed that TNF-α production in the dLN, analyzed by intracellular cytokine staining directly *ex vivo,* was restricted primarily to conventional T-cells (7.6% of total T cells) and to a lesser extent to NKT cells (5% of total NKT cells) (Fig. 5a). However, in terms of absolute cell number T cells are predominant producers for TNF-α in the dLN at 6 d.p.i. Both CD4^+^ and CD8^+^ T cells in the 6 d.p.i. dLN expressed TNF-α (i.e. ∼7–8% of the respective T-cells subset) (Fig. 5b). It is also noteworthy that the T-cells from the corresponding dLN of IAV-infected *il-21* ^−/−^ mice exhibited a significantly diminished protein expression of TNF-α compared to infected wild type mice both in terms of the percentage of each subset and absolute cell numbers (Fig. 5b). To further confirm the contribution of IL-21R signaling in TNF-α expression by IAV-activated T cells, mixed BM chimeras containing wild type (CD45.1^+^) and *il-21rα ^−/−^* (CD45.2^+^) BM in a 1∶1 ratio were generated. After 8 weeks, the successfully reconstituted mice were infected with A/PR/8/34 IAV. Since TNF-α expression can be regulated both pre-and post-transcriptionally, on 6 d.p.i. both wild type (CD45.1^+^) and *il-21rα ^−/−^* (CD45.2^+^) T cells (both CD4 and CD8 T cells) were isolated from the dLN by FACS and *tnf-α* gene expression in sorted T cells was determined by qPCR. As shown in figure.5c, *il-21rα ^−/−^* T cells (both CD4 and CD8 T cells) exhibited significantly diminished *tnf-α* gene expression compared to WT-T cells (Fig. 5c). Of note, IL-21 deficiency had no impact on TNF-α production by NKT cells in the 6 d.p.i. dLN of *il-21* ^−/−^ mice (data not shown). However, since NKT cells are the initial primary source of IL-21 in the 6 d.p.i. dLNs, IAV-infected *cd-1d ^−/−^* mice exhibited significantly diminished expression of TNF-α in conventional T cells compared to wild type mice at 6 d.p.i. (Fig. 5d). Finally, these data all together suggest that IL-21, initially produced by NKT cells, promotes TNF-α production by conventional T cells *via* IL-21R stimulation in the dLN of IAV-infected mice.

### TNF-α produced by T cells promotes CXCL9-mediated LAPC migration into the dLN and subsequent T_FH_ differentiation during IAV infection

**Figure 6:**
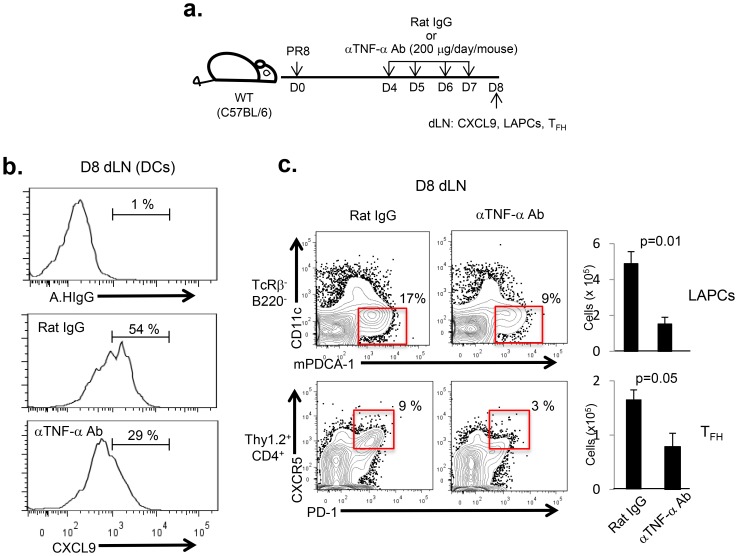
TNF-α promotes CXCL9-mediated LAPC migration into the dLN and subsequent T_FH_ differentiation during IAV infection. (**a**) C57BL/6 (WT) (n = 12) mice were infected with 0.05 LD_50_ of A/PR/8/34 virus. From 4 d.p.i. thru 7 d.p.i mice were treated (i.p.) with either isotype control Abs (Rat IgG) or αmTNF-α blocking mAb (XT3.11) (200 mg/day/mouse). At 8 d.p.i., (**b**) the expression level of CXCL9 was examined in DCs by FACS-analysis. (**c**) The extend of LAPC accumulation and T_FH_ differentiation in the dLN of infected mice following control Ab or αmTNF-α blocking Ab treatment were likewise evaluated by FACS-analysis. The data are presented as both percent population and absolute numbers (mean ± s.e.m.) and were analyzed by Student’s *t* test. Representative data from two independent experiments are shown.

**Figure 7:**
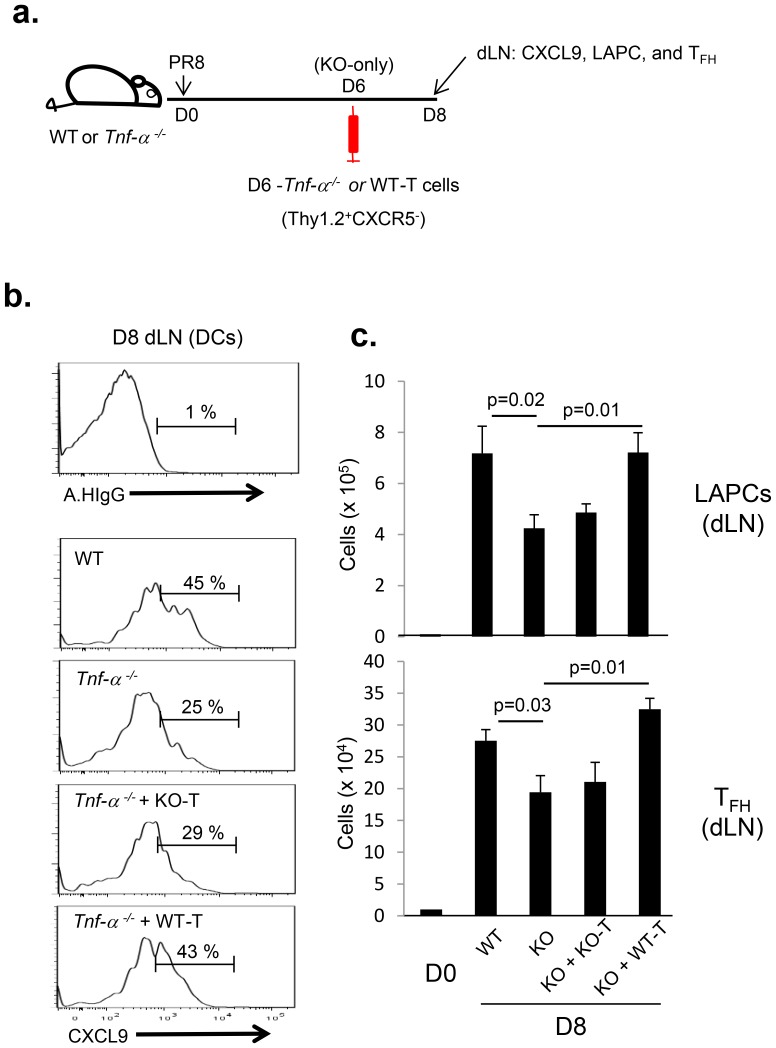
TNF-α producing T cells promotes CXCL9-mediated LAPC migration into the dLN and subsequent T_FH_ differentiation during IAV infection. (**a**) C57BL/6 (WT) (n = 12) and *tnf-α* ^−/−^ (KO) (n = 24) mice were infected with 0.05 LD_50_ of A/PR/8/34 virus. At 6 d.p.i. non-T_FH_ total T cells (Thy1.2^+^CXCR5^−^) were isolated from the dLN of A/PR/8/34 virus-infected either *tnf-α* ^−/−^ (KO-T) or C57BL/6 (WT-T) mice and adoptively transferred by the *i.v.* route (2×10^6^cells/mouse) into A/PR/8/34 infected *tnfα* ^−/−^ mice (KO). At 8 d.p.i., (**b**) the expression level of CXCL9 in DCs and (**c**) the extend of LAPC accumulation and T_FH_ differentiation in the dLN of recipient mice were examined and compared with that of both WT and KO mice by FACS-analysis. The data are presented as either percent population or absolute numbers (mean ± s.e.m.) and were analyzed by Student’s *t* test. Representative data from two independent experiments are shown.

In view of the above results it was of interest first to determine if TNF-α influenced the production of CXCL9 by DCs in the dLN and thereby the migration of LAPC from the infected lungs to the dLN and subsequent T_FH_ cell accumulation. To this end IAV-infected mice were treated by i.p. with either αmTNF-α neutralizing mAbs (XT3.11) or isotype control Abs (Rat IgG) over time frame including the initial migration of LAPC into the dLN i.e. between 4 d.p.i and 7 d.p.i. (Fig. 6a). The level of CXCL9 expression by dLN DCs was evaluated directly *ex vivo* 24 hrs later by flow cytometric analysis. *In vivo* neutralization of TNF-α resulted in a significant decrease in CXCL9 production by DCs (Fig. 6b). In parallel with the decrease in CXCL9 expression, we observed that the accumulation of both LAPC and T_FH_ cells in the dLN were significantly diminished in the IAV-infected mice following TNF-α neutralization as reflected in both the absolute numbers of these two cell types and their percentage within the dLN (Fig. 6c). Next, we examined whether IAV-infected *tnf-α* ^−/−^ mice displayed a phenotype comparable to that of mice in which TNF-αwas neutralized *in vivo* by neutralizing αmTNF-α mAb administration and whether the transfer of TNF-α producing T cells into *tnf-α* ^−/−^ mice could rescue the phenotype of these mice (Fig. 7a). Indeed, compared to wild type mice IAV-infected *tnf-α* ^−/−^ mice showed significantly diminished CXCL9 expression in DCs (Fig. 7b). The accumulation of both LAPC and T_FH_ cells in the dLN were also significantly diminished in the IAV-infected *tnf-α* ^−/−^ mice (Fig. 7c). Finally, the adoptive transfer of TNF-α producing non-T_FH_ total T cells (Thy1.2^+^CXCR5^−^) isolated from the dLNs of IAV-infected wild type mice at 6 d.p.i. (D6 WT-T, Fig. 5a) could rescue CXCL9 expression by DCs in *tnf-α* ^−/−^ mice. In addition, LAPC and T_FH_ accumulation in the dLNs were restored in *tnf-α* ^−/−^ mice following transfer of WT-T cells to levels comparable to that of IAV-infected wild type mice (Fig. 7b and 7c). However, the adoptive transfer of *il21rα* ^−/−^ T cells, which exhibit significantly diminished TNF-α expression compared to WT-T cells (Fig. 5c), could not rescue the phenotype in T_FH_ accumulation of *tnf-α* ^−/−^ mice (data not shown). Of note, by the repeated experiments, in which the donor wild type CD4 T cells (CD45.1^+^) were distinguished from recipient *tnf-α^−/−^* CD4 T cells (CD45.2^+^), we confirmed that the rescue of phenotype in T_FH_ accumulation of WT-T cell supplemented *tnf-α* ^−/−^ mice was due to the differentiation of recipient *tnf-α^−/−^* CD4 T cells into T_FH_ cells by the help from donor WT-T cells but not solely reflect T_FH_ differentiation from transferred donor WT-T cells (data not shown). Collectively, these results suggest that IL-21-induced TNF-α production from conventional T cells enhances T_FH_ differentiation in part at least *via* modulating CXCR3-CXCL9 dependent LAPC migration into the dLN during IAV infection.

## Discussion

IL-21, first identified as a product of activated human T cells, is a pleiotropic cytokine which has diverse effects on the immune response through its ability to modulate the activity of many immune cell types [23]–[25]. Primarily produced by activated CD4^+^ T cell (in particular, T_FH_ effector cells), IL-21 regulates B cell responses within the B cell follicular germinal center (GC) [25]. NKT cells are an additional potential major source of IL-21 and produce even higher level of this cytokine than activated conventional CD4^+^ T cells when appropriately stimulated [22]. As a result of engagement of IL-21Rα/c receptor complex, IL-21 promotes the survival and proliferation, as well as cytokine and chemokine production by multiple immune cell types including macrophages, B, T, NK and NKT cells [24].

Although CD4^+^ T_FH_ effector cells are the predominant cell type producing IL-21 during the germinal center response in the dLN, in this model of respiratory virus infection, we find that NKT cells likely are a major source of IL-21 during the early phase of CD4^+^ T_FH_ effector cell differentiation and GC formation in the dLN. Our unpublished data suggested that there is minimal IL–21 release or expression into the IAV infected lungs before day 5–6 post infection. Indeed, at later stage of infection IL–21 was present in the lung mostly derived from IAV-specific CD4^+^ T cells entering the infected lungs from the dLN. The stimulus for IL-21 production by the NKT cells responding to IAV infection in the dLN is not as yet defined. NKT cells have been reported to produce IL-21 following antigen receptor engagement or following stimulation by TLR ligands [17], [22], [25]. Since the A/PR/8/34 IAV strain employed in this analysis does not efficiently replicate in the dLN of infected mice, IL-21 production as a result of stimulation of TLR on NKT cells by IAV-derived TLR ligands generated in the dLN seems unlikely. The more likely possibility is that IL-21 is produced by NKT cells following TCR-engagement in response to recognition of lipid moieties released from IAV infected cells.

IL-21 was also initially proposed as an important T cell-derived soluble factor regulating T_FH_ differentiation through engagement of the IL-21R on recently activated CD4^+^ T cells prior to lineage commitment [8], [11], [13]. Subsequent reports [12], [32] including our findings herein demonstrating a reduced (by ∼ 50%) T_FH_ response in the dLN of IAV-infected *il-21rα* ^−/−^ mice (Fig. 1b) further substantiates the contribution of IL21 to T_FH_ differentiation. However, it was unclear whether IL-21 acts directly on naïve/recently activated CD4^+^ T cells to drive T_FH_ differentiation [4], [6], [12]. Indeed, our analysis of T_FH_ responses in mixed BM chimeric mice indicates that when evaluated in the presence of IL-21R signaling competent T cells IL-21R deficient responding CD4^+^ T cells are fully capable of undergoing T_FH_ differentiation (Fig. 1c). Therefore, during pulmonary IAV infection at least, there is no intrinsic signal delivered by IL-21 to the responding CD4^+^ T cells in the dLN which is required to program the cells along the T_FH_ differentiation pathway.

Our results suggest a novel and heretofore underappreciated role of IL-21 in regulating T_FH_ differentiation that is through the production of TNF-α. IL-21 either alone or in concert with other cytokines (i.e. IL-7 or IL-15) has been demonstrated to promote TNF-α production most notably from responding T cells [26]–[28]. We observed that during IAV infection that the absence of TNF-α resulted in a markedly diminished T_FH_ response (Fig. 6c and 7c). In addition, the major source of TNF-α in the responding dLN were T cells whose production of this cytokine required IL-21 and the expression of the IL-21R by the responding T-cells as defective signaling through the IL-21R resulted in significantly decreased TNF-α production by the T-cells (Fig. 5a, 5b and unpublished data).

TNF-α has been demonstrated to play a central role in stimulating chemokine expression at sites of inflammation including CXCL9, which is a strong chemotactic stimulus for mononuclear cells [29]–[31], [33]–[35]. We observed during IAV infection, that DCs are the major source of CXCL9 in the dLN (Fig. 4b) [16]. Of note, the absence of TNF-α mediated stimulation significantly but not completely diminished CXCL9 production by DCs isolated from the dLN of IAV-infected mice (Fig. 6b and 7b). This incomplete inhibition of CXCL9 expression may due to an effect of other soluble factors present in the dLN which are capable of regulating CXCL9 expression in the dLN, most notably, IFN- [34], [35]. CXCL9 production by the dLN DCs was also significantly decreased in IAV-infected IL-21 or IL-21R deficient mice (Fig. 4c and 4d). However, in our mixed BM chimera study the absence of IL-21R expression on the dLN DC had no direct effect on CXCL9 production by these cells suggesting that the impact of IL21/IL21R signaling on CXCL9 production by the dLN DC was indirect (Fig. 4e), that is, through the effect of IL-21 on TNF-α production. Even though we cannot rule out the possibility that TNF-α works synergistically with IFN- in the dLN to induce CXCL9 expression from DCs [34], [35], it is unlikely that IL-21 also modulates IFN- expression since both *il-21* ^−/−^ and *il-21rα* ^−/−^ mice showed no difference in IFN- gene expression in the dLN post-IAV infection (unpublished data).

We believe that the likely link between IL-21/IL-21R signaling, TNF-α production and T_FH_ differentiation is *via* LAPC. This antigen presenting cell type picks up vial antigen in the IAV-infected lungs, migrate from the lungs into the dLN late in the infection cycle (i.e. between 6 and 12 d.p.i.) where these cells facilitate T_FH_ differentiation of Ag-activated CD4^+^ T cells. We recently reported that the migration of LAPC into the inflamed dLN is largely CXCR3-CXCL9 dependent [16]. In the current report we find that both IAV infected *il-21* ^−/−^ and *il-21rα* ^−/−^ mice showed significantly diminished (∼ 60–70%) LAPC migration/accumulation into the dLN (Fig. 3a and 3b) and concomitantly a decreased T_FH_ response following IAV infection in spite of normal CXCR3 expression by the LAPC from the IL-21/IL-21R signaling defective IAV-infected mice (Fig. 1b and 4a). Most important, the absence of TNF-α mediated stimulation *in vivo* during IAV infection, like defective IL-21/IL-21R signaling, resulted in both decreased LAPC migration into the dLN and in a diminished T_FH_ response (Fig. 6c and 7c). IL-21 also modulates B cell response which contributes to the maintenance of T_FH_ response [6]. Although B cells are poor inducers for initial T_FH_ differentiation [16], we cannot rule out the possibility that at the maintenance phase of T_FH_ response during IAV infection (after 9–10 d.p.i.) IL-21, predominantly produced by T_FH_ cells, may also contribute to T_FH_ maintenance *via* modulating B cells. Although IL–21, TNF-α and CD1d are implicated in anti-IAV T_FH_/GC-B cell response, we found that deficiency in IL–21 or IL–21R has no effect on virus clearance or recovery from IAV infection in C57BL/6 background (unpublished data). Also, as we reported *cd-1d* deficiency does impact on IL–5 production during IAV infection but does not affect virus clearance or recovery from IAV infection in BALB/c background [36].

In conclusion, in this report we have identified a novel mechanism, by which IL21 promotes optimal T_FH_ responses in pulmonary virus infection. Our results suggests that during IAV infection IL-21 produced in the dLN early in infection i.e. 5–6 d.p.i. (most likely by NKT cells in the dLN) stimulates TNF-α production by CD4^+^ and CD8^+^ T cells responding to infection in the dLN. The TNF-α stimuli enhances CXCL9 production by dLN resident DCs which in turn acts as a chemotactic stimulus to promote LAPC migration into the dLN. As a result of the increased accumulation of LAPC, this antigen presenting cell can facilitate optimal differentiation of Ag-activated responding CD4^+^ T cells in the dLN into T_FH_ cells.
